# Epidemiology and risk factors of community-acquired pneumonia in patients with different causes of immunosuppression

**DOI:** 10.1007/s15010-024-02314-w

**Published:** 2024-06-27

**Authors:** Fabian Reichel, Falko Tesch, Saskia Berger, Martin Seifert, Dirk Koschel, Jochen Schmitt, Martin Kolditz

**Affiliations:** 1 Medical Department I, Division of Pneumology, University Hospital Carl Gustav Carus, TU Dresden, Dresden, Germany; 2 East German Lung Center / Ostdeutsches Lungenzentrum Dresden-Coswig, Coswig, Germany; 3 Center for Evidence-Based Healthcare, University Hospital and Faculty of Medicine Carl Gustav Carus at TU Dresden, Dresden, Germany; 4 Hospital Pharmacy, University Hospital Carl Gustav Carus, Dresden, Germany; 5 Department of Internal Medicine and Pneumology, Fachkrankenhaus Coswig, Coswig, Germany

**Keywords:** Community-acquired pneumonia, Immunosuppression, Steroids, Risk, Hospitalization, Mortality

## Abstract

Immunosuppression constitutes a significant risk for community-acquired pneumonia (CAP). Nevertheless, specific causes of immunosuppression and their relevance for incidence, etiology and prognosis of CAP are insufficiently investigated.

We conducted a population-based cohort study within a statutory health insurance in Germany from 2015 to 2018. CAP was retrieved by ICD-10-GM codes. Episodes of immunosuppression were identified by coded conditions (hematologic neoplasms, stem cell or organ transplantation, neutropenia, HIV, primary immunosuppressive syndromes) or treatments (immunosuppressants, antineoplastic drugs, systemic steroids). Endpoints were defined as occurrence of CAP (primary), hospitalization, 30-day mortality and CAP associated with rare pathogens. Our analysis utilized the Andersen-Gill model adjusted for sex, age, level of long-term care, vaccination status, community type and comorbidities.

942,008 individuals with 54,781 CAPs were included (hospitalization 55%, 30-day mortality 14.5%). 6% of individuals showed at least one episode of immunosuppression during the study period with systemic steroids (39.8%) and hematologic neoplasms (26.7%) being most common. Immunosuppression was recorded in 7.7% of CAPs. Besides classical risk factors such as age and level of long-term care, immunosuppressed patients were most prone to CAP (HR 2.4[2.3–2.5]) and consecutive death (HR 1.9[1.8–2.1]). Organ and stem cell transplantation (HR 3.2[2.6–4.0] and 2.8[2.1–3.7], respectively), HIV (HR 3.2[1.9–5.4]) and systemic steroids (> 20 mg prednisone daily dose equivalent (HR 2.7[2.4–3.1])) showed the highest risk for contracting CAP. CAP by rare pathogens was strongly associated with immunosuppression (HR 17.1[12.0–24.5]), especially HIV (HR 34.1[7.6–153]) and systemic steroids (HR 8.2[4.6–14.8]).

Our study elucidates the relevance of particular immunosuppressive conditions including systemic steroids for occurrence and prognosis of CAP.

**Supplementary Information:**

The online version contains supplementary material available at 10.1007/s15010-024-02314-w.

## Introduction

Community-acquired pneumonia (CAP) is associated with a significant healthcare burden in elderly and multimorbid patients. This association is specifically modulated by the presence of immunosuppression, which is linked to not only a higher CAP incidence and severity, but also to rare pathogens not covered by empirical treatment [1–4]. However, there is still no widely accepted definition of immunosuppression [1, 3, 5] as causes of immunosuppression are complex and include both innate and acquired medical conditions as well as numerous treatments resulting in immune impairments. The number of people at risk is presumably rising due to improved survival of patients with immunosuppressive conditions and increasing implementation of immune-modulating treatments for a variety of diseases. Earlier, most studies estimated that 2 to 4% of western adult populations are immunosuppressed [6–10], but a recent study reported an increase to 6.6% within the last decade [11]. Due to the complexity of immunosuppression data often lacks precision. In hospitalized patients with CAP, studies reported up to 18–25% immunosuppression [12, 13], however population-based non-selected data including in- and outpatients along with detailed information on different immunosuppressive conditions are unavailable. In order to target preventive measures like vaccines or drug prophylaxis and to potentially address modifiable immunosuppressive conditions in patients at risk, such information is key. Therefore, we performed a large population-based study to evaluate the specific risk of different immunosuppressive conditions in order to identify risk factors for occurrence and outcome as well as association with rare pathogens in adult in- and outpatients with CAP.

## Methods

### Study population

We conducted a retrospective cohort study using data provided by AOK PLUS, the most common statutory health insurance in the German federal state of Saxony holding contracts with about 50% of the state’s population. Provided pseudonymized data consisted of detailed information about sociodemographics, diagnoses, inpatient and outpatient treatment, performed procedures, prescribed drugs and medical remedies. Data was retrieved 2015–2018 for CAP epidemiology and 2010–2018 for vaccination status considered in the covariate analysis. Patients were included when alive and at legal age (> 17 years) in 2015 and continuously insured (> 349 days per year) by AOK PLUS between 2010 and 2014. This analysis was approved by the Saxon State Ministry for Social Affairs and the ethics committee of TU Dresden (EK 143052018).

### Outcome definition

Coded diagnosis of CAP during the study period was declared to be the primary endpoint of our investigation. We secondarily examined for coded diagnosis of CAP associated with rare pathogens, the need for hospitalization and 30-day all-cause mortality after diagnosis of CAP. The risk of meeting the endpoint criteria was calculated for below described factors and covariates.

We identified CAP cases using ICD-10-GM codes (International Classification of Diseases, 10th Revision, German Modification) as recently published [4]. CAP was defined as hospitalization and a main discharge diagnosis of pneumonia coded by ICD-10-GM A31.0, A42.0, A43.0, A48.1, A70, B01.2, B25.0, B44.0, B44.1, B45.0, B46.0, B59, B58.3, J10.0, J11.0, J12.x–J16.x, J18.x, J69.0, J85.1 or hospitalization and a main discharge diagnosis of sepsis (A40.x–A41.x) in combination with a secondary diagnosis of pneumonia as encoded above or an ambulatory diagnosis of pneumonia as previously defined followed by a prescription of an anti-infective drug (ATC J01, J02A, J05) [4].

CAP associated with rare pathogens was classified as a subgroup of above defined CAP when encoded as A31.0, A42.0, A43.0, B01.2, B25.0, B44.0, B44.1, B45.0, B46.0, B59 or B58.3.

Mortality was defined as death of any cause within a 30-day period after diagnosis of CAP.

### Definition of immunosuppression

Immunosuppression can be obtained by both an underlying condition and a drug-induced deficiency. As these characteristics can vary by time for an individual person, we considered episodes of immunosuppression.

Underlying immunosuppressive conditions were defined as (1) active hematologic neoplasm, (2) stem cell or solid organ transplantation, (3) neutropenia, (4) HIV infection or (5) defined primary or humoral immune deficiency syndromes; the corresponding ICD-10-GM codes are depicted in Table S1. An immunosuppressive condition was considered as existent from the first day of inpatient documentation or first quarter of outpatient documentation.

For drug-induced immunodeficiency we established four definitions: (I) antineoplastic drugs including rituximab (Table S2), (II) immunosuppressants (Table S3), (III) systemic steroid therapy for more than 90 days using a prednisone daily dose equivalent (PDDE) of 10–20 mg (Table S4), (IV) systemic steroid therapy for more than 30 days using a PDDE of > 20 mg (Table S4). We included immunosuppressive medication when prescribed at least twice within one or two consecutive quarters of a year. For definition I immunosuppressive exposure started immediately after the first prescription and ended 90 days after the last one. Exposure to immunosuppression according to definitions II-IV began 14 days after the first drug prescription and ended 90 days after the last one. In case of a period without prescription for > 90 days the immunocompromised state ceased as stated above and was reconsidered 14 days after a following prescription. Episodes with singular steroid prescriptions equivalent to > 10 mg prednisone were excluded from the analysis since they could not be assigned to either definition III or IV as the exposure was unclear.

### Statistical analysis

Since our primary endpoint CAP could occur multiple times during the observation period in each person the Andersen-Gill model was used for our cohort study [14]. It allows the analysis of recurrent event episodes of various lengths over the study period. Patients’ episodes were split each time a new diagnosis emerged or a relevant change in medication occurred. All CAPs diagnosed within 30 days in a single patient were considered the same episode. Estimates were displayed as hazard ratios (HR) and their corresponding 95% confidence intervals (CI) and cumulative hazard rates were plotted for subgroups.

The analysis was adjusted for the covariates: age, sex, level of long-term care (long-term care as defined by German Social Code XI), type of community (cities > 85,000 inhabitants holding Saxony’s largest healthcare providers including all of the state’s maximum-care hospitals compared to provincial/rural areas), pneumococcal vaccination up to 5 years prior to study period, influenza vaccination up to 1 year prior to study period and chronic comorbidities. The latter were defined and grouped according to ICD-10-GM codes as shown in Table S5 that were adapted from comorbidity measurement tools developed by Charlson and Elixhauser [15]. For internal case validation comorbidities had to be coded twice or more to be considered a relevant diagnosis [16]. More than three consultations were required for a cancer diagnosis to be considered [17]. The analyses were performed with R (v3.6.3) and RStudio (v2023.09.1 + 494) using the survival package, the survminer package for plotting the absolute risks and the forestplot package to create forest plots [18, 19].

## Results

### Occurrence of CAP and immunosuppression

**Fig. 1:**
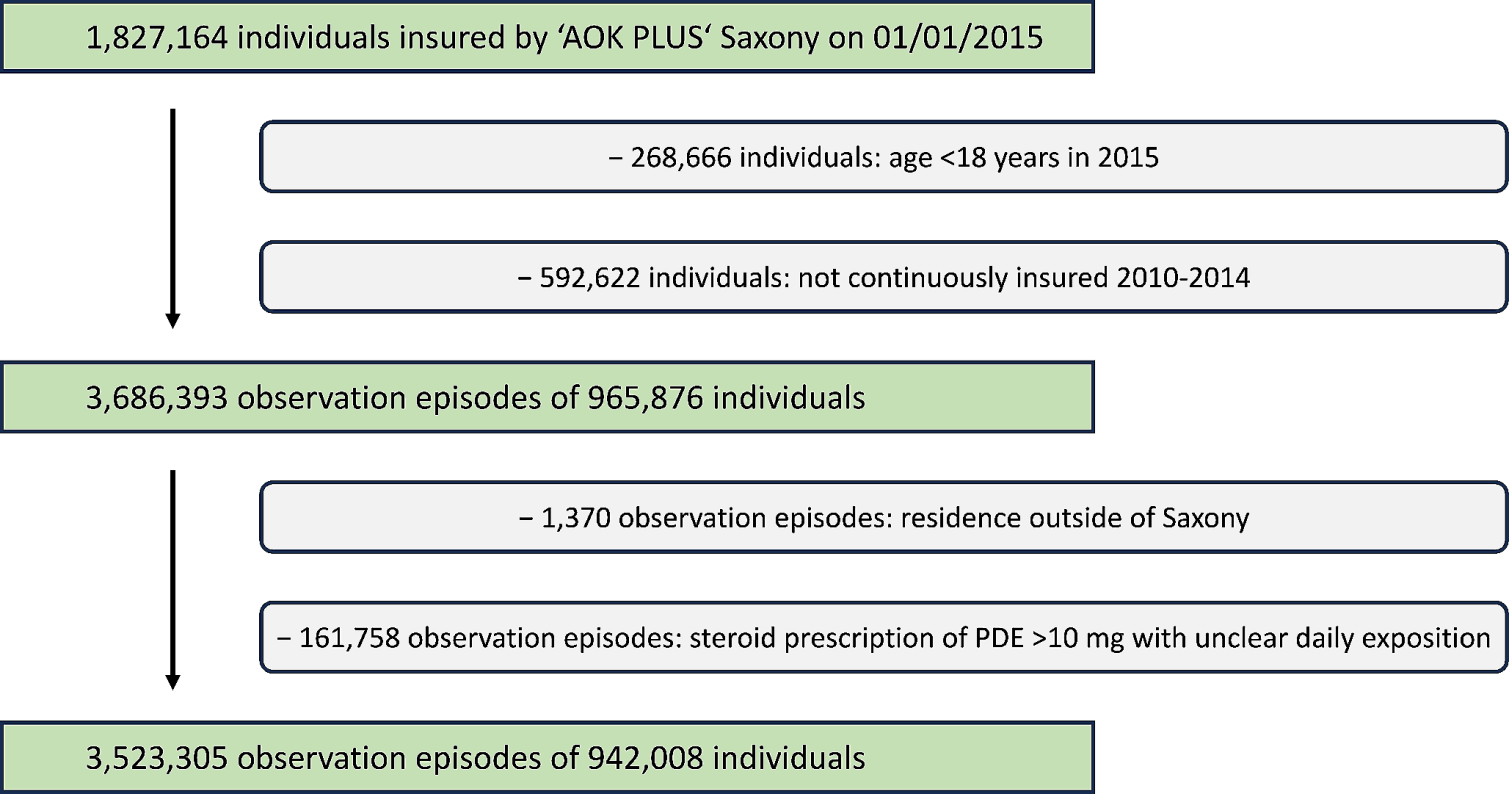
Flowchart of selection process of study population. After applying the listed inclusion and exclusion criteria the observation group consisted of 942,008 individuals providing a total of about 3.5 Mio. observation episodes. PDE – prednisone dose equivalent

We acquired 1,827,164 insured people with AOK PLUS on the first day of 2015 (Fig. 1). We excluded people that were underage or not continuously insured by AOK PLUS and omitted episodes regarding immunosuppressive status of (1) people living outside of Saxony or (2) with steroid prescriptions of unclear daily dose as mentioned above. As a result, a total of 942,008 people with 3,523,305 episodes with or without immunosuppression were included in our study; their characteristics are shown in Table 1.

**Table 1:** Characteristics of study population

	No. of individuals	Fraction [%]
Total study population	942,008	100
Male	419,754	44.6
Female	522,254	55.4
Year of birth ≥1980	144,850	15.4
Year of birth 1970–79	85,263	9.1
Year of birth 1960–69	131,395	13.9
Year of birth 1950–59	174,291	18.5
Year of birth 1940–49	156,011	16.6
Year of birth 1930–39	171,282	18.2
Year of birth < 1930	78,916	8.4
Mean age in 2015	60.09 (SD 19.68)	
CAP once	39,141	4.2
CAP more than once	6,474	0.7
CAP by rare pathogen once	252	< 0.1
CAP by rare pathogen more than once	102	< 0.1
Cardiovascular disease	277,374	29.4
Neurological disorder	236,672	25.1
Pulmonary disease	144,980	15.4
Renal disease	150,125	15.9
Gastrointestinal disease	116,958	12.4
Diabetes mellitus	267,387	28.4
Rheumatic disease	53,567	5.7
Solid tumor w/o immunosuppression	99,748	10.6
Immunosuppression in general	56,785	6.0
Immunosuppressive condition		
Active hematologic neoplasm	15,192	1.6
Solid organ transplantation	1,364	0.1
Stem cell transplantation	357	0.0
Neutropenia	4,218	0.4
HIV-infection	529	0.1
Primary and humoral immune deficiency	3,178	0.3
Pharmaceutic immunosuppression (I): antineoplastic drugs	13,337	1.4
Antineoplastic drugs w/o rituximab	13,202	1.4
Rituximab	697	0.1
Pharmaceutic immunosuppression (III): PDDE 10–20 mg	14,039	1.5
Pharmaceutic immunosuppression (IV): PDDE > 20 mg	11,308	1.2
Pharmaceutic immunosuppression (II): immunosuppressants	13,337	1.4
Methotrexate	5,574	0.6
Azathioprine	2,257	0.2
TNF inhibitors	1,322	0.1
Mycophenolate	1,142	0.1
Leflunomide/teriflunomide	1,091	0.1
Interleukin inhibitors	870	0.1
Mycophenolate plus mTOR- or calcineurin inhibitors	870	0.1
Calcineurin inhibitors	843	0.1
Abatacept/belatacept	316	0.0

We identified 56,785 individuals (6.0% of the total cohort of all continuously insured individuals) facing at least one episode of immunosuppression throughout the study period. The majority of people with an underlying immunosuppressive condition suffered from active hematologic neoplasms (15,192; 26.8%) while steroid therapy was most common in patients with drug-induced immunosuppression (22,587; 39.8%).

Among the study population 45,615 people faced a total of 54,781 CAPs. In 6,474 patients (14.2% of all CAP patients) the diagnosis was noted more than once during the study period. We found a hospitalization rate of 54.6%. Overall, 30-day mortality was at 14.5%.

Amid all CAPs 7.7% (4,211) suffered from an underlying immunosuppression. Active hematologic neoplasms were the most frequent cause (2,168; 51.5%) followed by systemic steroid therapy (740; 17.6%), immunosuppressants (734; 17.4%) and antineoplastic drugs (652; 15.5%).

Of all CAPs 630 (1.2%) were associated with rare pathogens according to ICD-10-coding and therefore not covered by the standard empirical antimicrobial therapy (Table 2). Despite a higher median year of birth of 1951 (compared to 1938 for all CAPs), admission rate was similar to regular pneumonia with 57.1% of patients hospitalized while the proportion of immunocompromised patients was considerably increased by more than a fourfold (36%). All-cause 30-day mortality was 8.9%.

**Table 2:** Occurrence of CAP associated with rare pathogens according to ICD-10-GM codes

ICD-10-GM Code	Corresponding diagnosis	No. of coded diagnoses	No. of CAPs with > 1 coded CAP by rare pathogen^a^
A31.0	Lung infection by NTM	176	23
A42.0	Actinomycosis of the lung	44	1
A43.0	Pulmonary nocardiosis	3	
B01.2	Varicella pneumonia	1	
B25.0	Pneumonia by cytomegalovirus	26	3
B44.0	Invasive pulmonary aspergillosis	55	7
B44.1	Other pulmonary aspergillosis	220	21
B45.0	Pulmonary cryptococcosis	1	
B46.0	Pulmonary mucormycosis	1	
B58.3	Pulmonary toxoplasmosis	1	
B59	Pneumocystis pneumonia	134	9
**total**		662	64

^a^ in 32 cases there were two codes for CAP associated with rare pathogens within a single episode. 15 times a combination of A31.0 and B44.1, 8 times A31.0 and B59, 4 times B44.0 and B44.1, 3 times B44.0 and B25.0, once B44.1. and A42.0, once B44.1 and B59

### Immunosuppression as risk factor for CAP occurrence, hospitalization and mortality

**Fig. 2:**
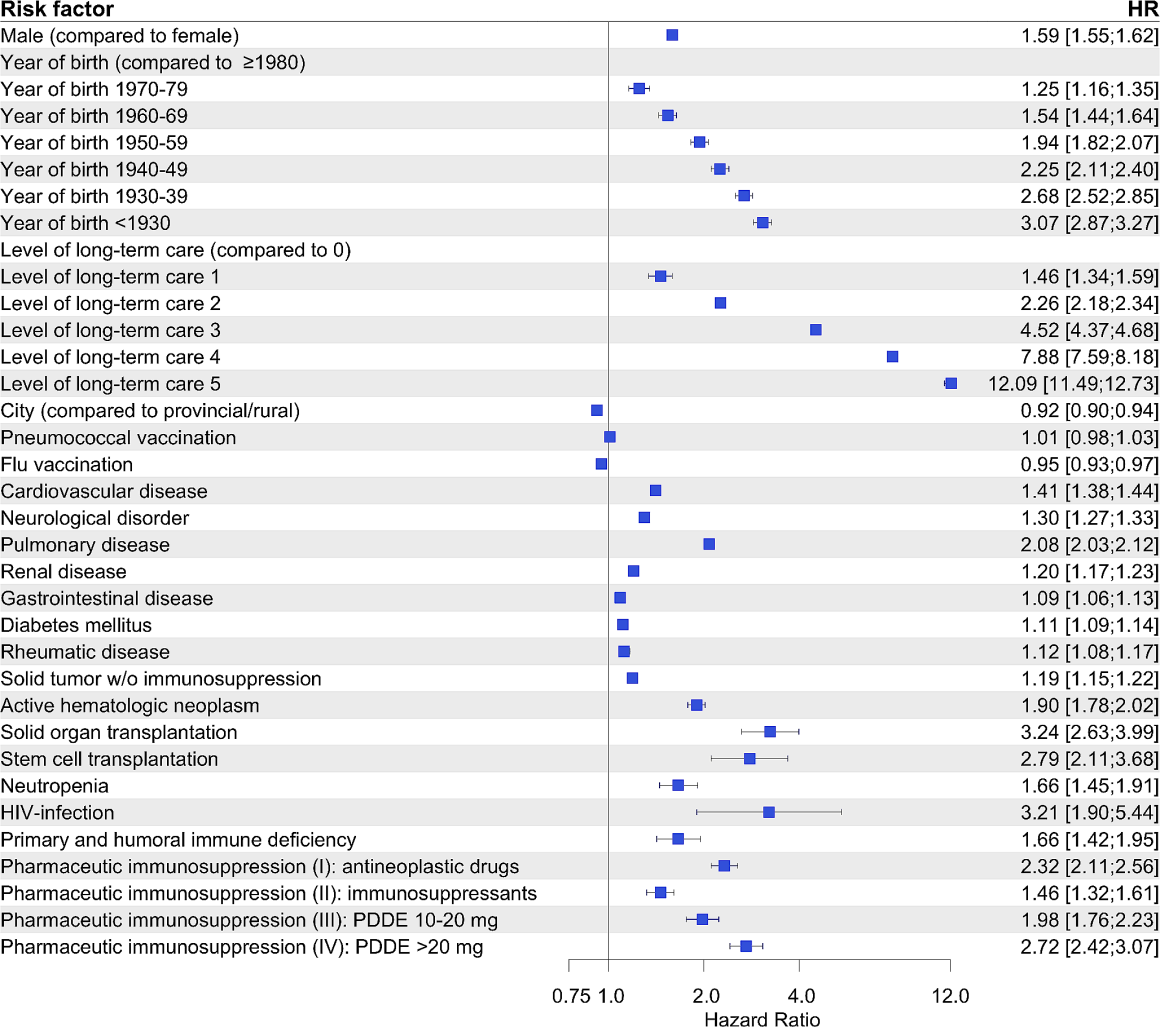
Risk factor evaluation for occurrence of CAP. 54,781 CAPs out of ∼ 3.5 Mio. total recorded observation episodes were analyzed. Hazard ratios (HR) and corresponding 95% confidence intervals were calculated using the Andersen-Gill model and are displayed as forest plot. PDDE – prednisone daily dose equivalent

Figure 2 demonstrates the risk factor evaluation for CAP including all predefined immunosuppressive conditions after multivariable analysis. Increasing age and rising levels of long-term care contributed most to contracting CAP.

**Fig. 3:**
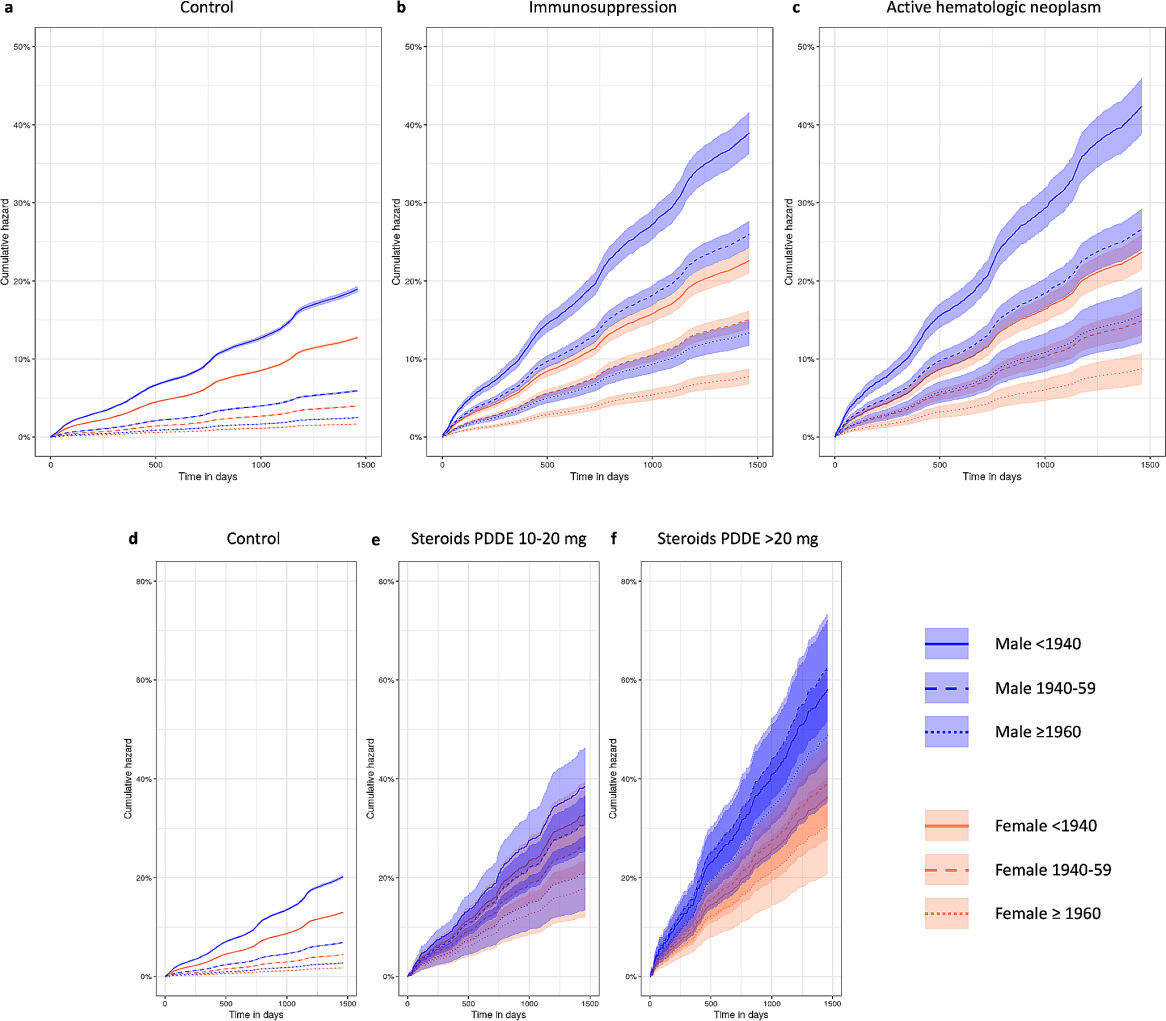
Absolute risks for CAP under specific immunosuppressive conditions. Patients with immunosuppression in general (**b**), with an active hematologic neoplasm (**c**) and systemic steroid therapy of PDDE 10–20 mg (**e**) or PDDE >20 mg (**f**) compared to controls ((**a**) and (**d**)) that are not subject to the respective risk factor. Cumulative hazards and corresponding 95% confidence intervals are given depending on sex and age. PDDE – prednisone daily dose equivalent

Aside from chronic pulmonary comorbidities an immunocompromised state was the most critical risk factor among medical conditions for CAP (HR 2.4 [95%-CI 2.3–2.5] for any kind of immunosuppression). In particular, we found solid organ transplantation (HR 3.2) and HIV infection (HR 3.2) to be highly associated with CAP, followed by stem cell transplantation (HR 2.8), systemic steroid therapy using PDDE > 20 mg (HR 2.7) and antineoplastic drugs (HR 2.3) (Fig. 2). Selected absolute risk rates for CAP according to age, sex and immunosuppressive states are shown in Fig. 3.

**Fig. 4:**
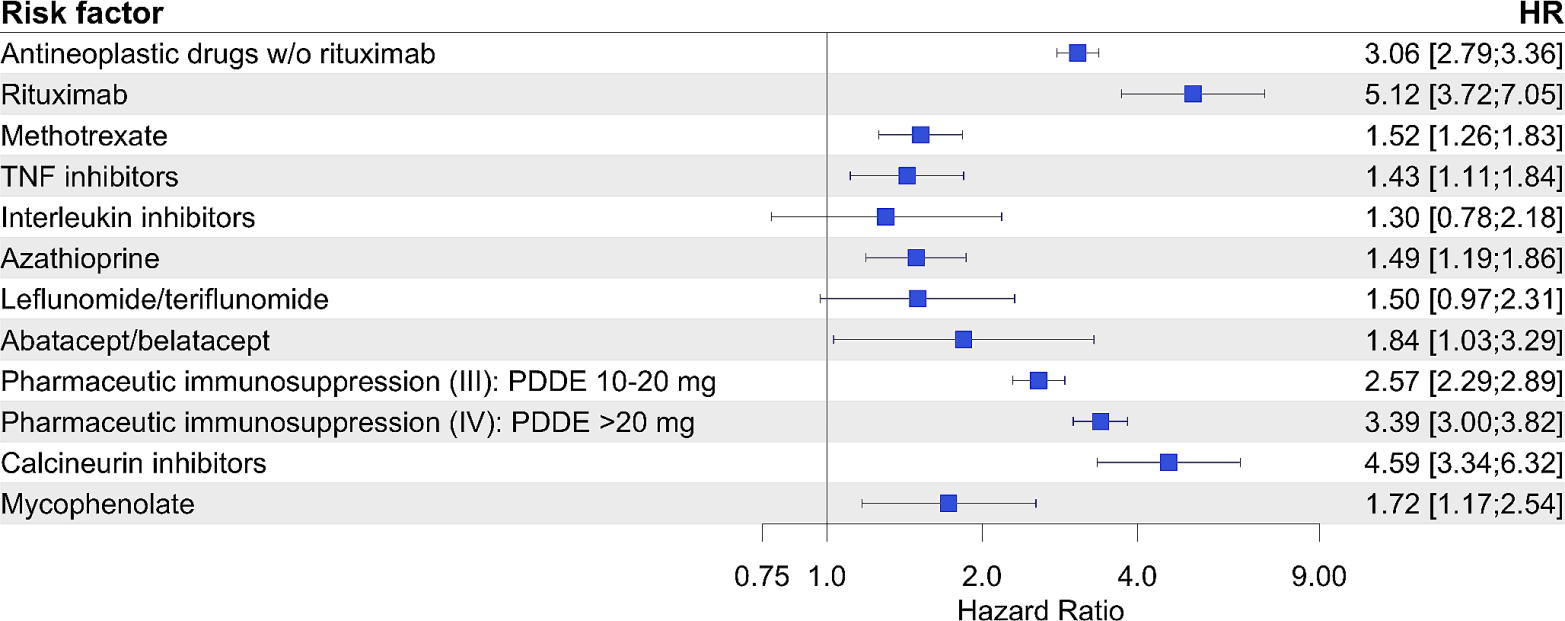
Risk factor evaluation for occurrence of CAP under common immunosuppressants. Risks were additionally adjusted for age, level of long-term care, vaccination status and type of community. 54,781 CAPs out of ∼ 3.5 Mio. total recorded observation episodes were analyzed. Hazard ratios (HR) and corresponding 95% confidence intervals were calculated using the Andersen-Gill model and are displayed as forest plot. PDDE – prednisone daily dose equivalent

Figure 4 shows a specific evaluation of the most frequent immunosuppressive drugs corrected for sex, age, level of long-term care, type of community and vaccination status. Rituximab and calcineurin inhibitors accounted for the highest CAP risks (HR 5.1 and HR 4.6, respectively), followed by systemic steroid therapy using PDDE > 20 mg (HR 3.4).

To further assess the specific relative risks of particular immunosuppressive drugs we performed an additional analysis considering solely patients on immunosuppressants including steroids (definitions of immunosuppressive medications II-IV) (*n* = 928). We set methotrexate treatment as control (HR 1) and corrected for the above-named covariates (Fig. S1). Compared to methotrexate, we identified the highest risk for calcineurin inhibitors (HR 4.6), closely followed by a steroid treatment using PDDE > 20 mg (HR 4.3).

Evaluation of risk factors for hospitalized CAP is presented in Fig. S2. People at ≥75 years of age were at a vastly higher risk of requiring inpatient treatment (HR > 7). The hospitalization rate more than doubled in immunosuppressed patients (HR 2.5 [95%-CI 2.4–2.6]). Organ and stem cell transplant receivers were most likely to require inpatient treatment (HR 3.5 and HR 3.6, respectively), followed by a systemic steroid therapy of PDDE > 20 mg (HR 2.7). All-cause 30-day mortality was extensively elevated in patients with an intensive level of long-term care (HR > 70) (Fig. S3). Nevertheless, it was also particularly prominent among immunocompromised individuals (HR 1.9 [95%-CI 1.8–2.1]). In fact, it was dominated by stem cell transplantation (HR 5.5). Antineoplastic drugs (HR 2.8) und solid organ transplantation (HR 2.5) likewise indicated substantial hazards.

### Immunosuppression as risk factor for CAP associated with rare pathogens

**Fig. 5:**
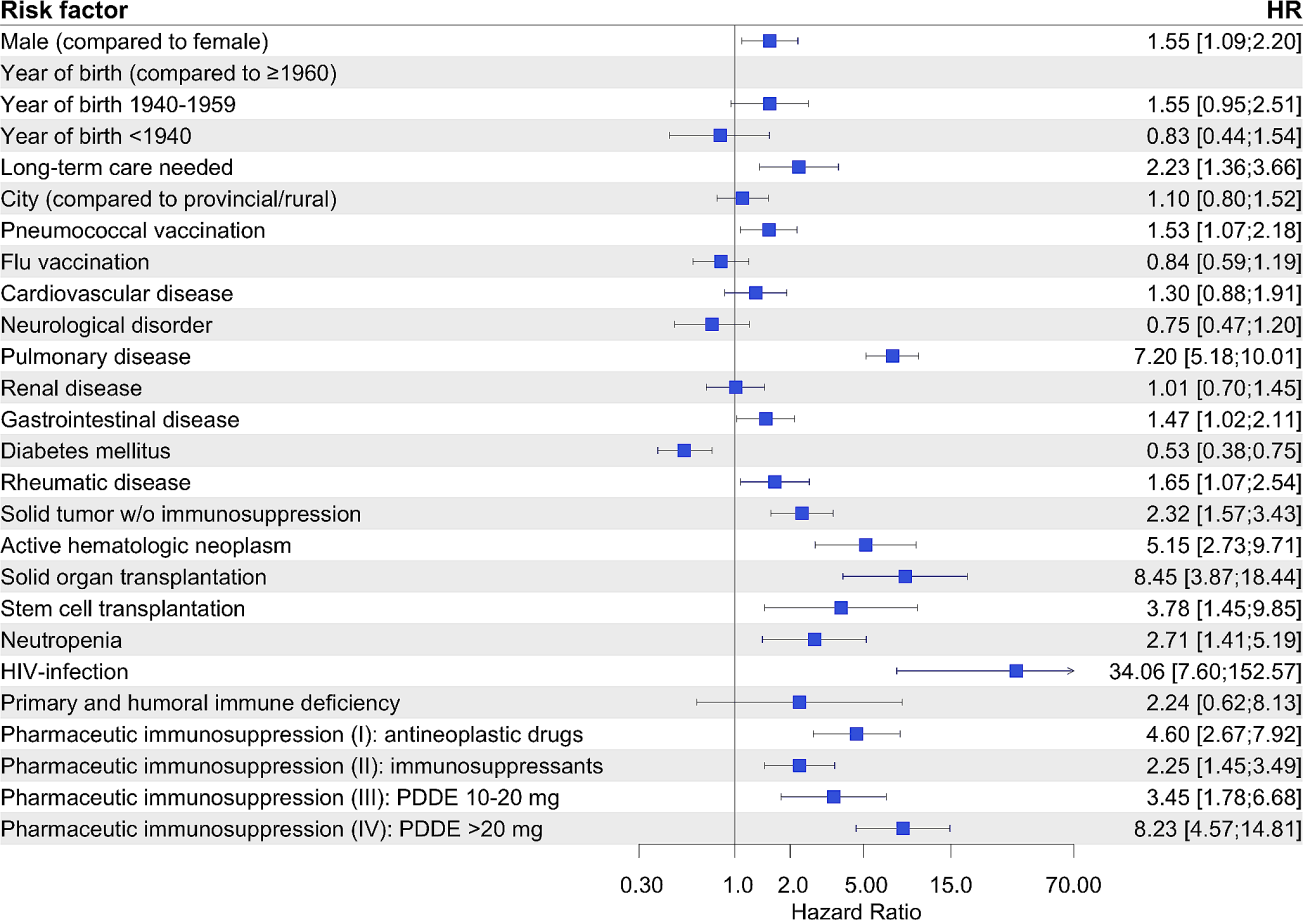
Risk factor evaluation for occurrence of CAP associated with rare pathogens. 630 CAPs with ICD-10-coded rare pathogen association out of ∼ 3.5 Mio. total recorded observation episodes were analyzed. Hazard ratios (HR) and corresponding 95% confidence intervals were calculated using the Andersen-Gill model and are displayed as forest plot. PDDE – prednisone daily dose equivalent

Among the 630 recorded CAPs associated with rare pathogens by ICD coding *Aspergillus spp.* (275; 43.7%), *nontuberculous mycobacteria* (NTM) (176; 27.9%) and *Pneumocystis jirovecii* (134; 21.3%) were most common (Table 2). We evaluated risk factors of contracting CAP associated with rare pathogens compared to the entire study population (Fig. 5).

Immunosuppression in general was present in 36% of the 630 cases making it the most substantial risk factor (HR 17.1 [95%-CI 12.0–24.5]), while age and level of long-term care played a limited role only. A pre-existing lung disease, however, was accountable for a HR of 7.2. Among immunosuppressive conditions active hematologic neoplasms depicted the most common condition (102 cases, 45%), but people suffering from HIV (HR 34.1) or receiving a systemic steroid treatment using PDDE > 20 mg as well as patients after solid organ transplantation faced the highest risk of contracting rare pulmonary infections (HR 8.2 and HR 8.5, respectively). Though we did see a higher risk for rare pathogen-associated CAP for solid tumors lacking additional immunosuppressive treatment (HR 2.3), its risk seems far less relevant when compared to other immunocompromising factors.

Immunosuppression also leads to an extensively elevated hospitalization rate for CAP associated with rare pathogens (HR 17.6 [95%-CI 12.6–24.6]). HIV (HR 20.1) and solid organ transplantation (HR 13.3) were the most precarious risk factors closely followed by a systemic steroid therapy which suggested a dose dependency (HR_PDDE 10−20mg_ 2.2; not significant and HR_PDDE >20mg_ 8.4, respectively) (Fig. S4). Additionally, immunosuppressed patients faced the highest 30-day mortality after CAP associated with rare pathogens (HR 31.6 [95%-CI 15.7–63.6]). Solid organ transplantation (HR 8.8) and neutropenia (HR 8.2) dominated the risk for CAP related death within 30 days when associated with rare pathogens (Fig. S5).

## Discussion

To the best of our knowledge, this study conducted an extensive investigation into immunosuppression associated with CAP, analyzing one of the largest populations ever examined, encompassing both common and rare pathogens. The main findings of our population-based representative study are as follows: (1) a high proportion of 6% of the study population had at least one episode of immunosuppression and 7.7% of ambulatory and hospitalized all-cause CAPs occurred under immunosuppression; (2) apart from age and high level of long-term care, immunosuppression was the most relevant factor related to CAP and CAP-associated mortality; (3) different immunosuppressive conditions caused different hazards with hematologic neoplasms, solid organ and stem cell transplantation, HIV infection as well as prescriptions of high-dose systemic steroids, rituximab or calcineurin inhibitors showing the highest risk, and (4) immunosuppression is particularly relevant for CAP associated with rare pathogens in which HIV infection, solid organ or stem cell transplantation and prescription of high dose systemic steroids were dominant risk factors.

We found 6% of the population to have at least one episode of immunosuppression during the 4-year observation period. Earlier investigations identified a lower prevalence of up to 4% which might be caused by less rigorous drug evaluation, a true increasing prevalence, or our lengthened observation period [7–10]. A just recently published American study virtually mirrored our findings giving a prevalence of 6.6% suporting a potential increase of immunosuppressed patients over the past years [11]. A universal definition of immunosuppression is still lacking, and specification substantially depends on the authors’ definitions. There have been divergent views on whom is considered immunocompromised especially when it comes to solid tumors without additional immunosuppressive therapy, as previously addressed [3, 5, 8–10, 12]. The data we provided demonstrates that patients with solid tumors in absence of immunocompromising drugs are at only moderately elevated risk of contracting CAP (HR 1.2) (Fig. 2). Furthermore, they show a notably lower risk (HR 2.3) of becoming infected by rare pathogens when compared to evidently immunosuppressed patients (HR 17.1). Recently an expert workshop of the American Thoracic Society confirmed that patients with solid tumors quickly recovering from a possible short-term neutropenia without exposure to immunosuppressive drugs are not to be considered immunosuppressed [1].

Along with past publications we found active hematologic neoplasms to be the most prevalent underlying immunosuppressive condition and systemic steroid therapy the most common immunosuppressive treatment [6, 10, 12].

Immunosuppression entails a compelling risk of contracting CAP alongside classic risk factors like age, level of long-term care and comorbidities [1, 2, 4, 13]. At HR 2.4 immunosuppression was the leading risk factor of all underlying conditions even exceeding the previously known high risk of chronic lung diseases which we calculated at HR 2.1. While immunosuppression was found in 7.7% of all CAPs, Di Pasquale et al. found it to be disproportionally present in 17.9% of hosptalized German CAP patients that were reported to their study (31/173), highlighting its risk for inpatient treatment which we calculated at HR 2.5. Additionally, immunosuppression led to a significant increase in all-cause 30-day-mortality (HR 1.9) dominating all other chronic comorbidities [2, 4, 13].

With just under one million included patients among which > 50,000 suffered from immunosuppression we added detailed data on differential hazards of particular immunocompromising conditions. We found systemic steroid exposure with PDDE ≥ 10 mg to be the most frequent drug-induced immunocompromising cause affecting 2.7% of the complete cohort. Regarding short-term use that we intentionally excluded Waljee et al. identified a remarkable 21.1% of > 1.5 Mio. American privately insured outpatients that were exposed to oral steroids over a study-period of 3 years. Even short-term use put patients at fairly higher risk to suffer from sepsis (IRR 5.3 [95%-CI 3.8–7.4]) and other complications [20]. With respect to respiratory diseases Fardet et al. uncovered lower respiratory tract infections at HR 5.42 (95%-CI 5.2–5.6) in patients on systemic steroids for > 15 days [21]. This matches our data, where systemic steroids above PDDE of 20 mg displayed an independent HR of 3.4 for CAP, subsequent only to that of strong immunosuppressors like calcineurin inhibitors or rituximab (Fig. 4). This was further underlined by its higher risk association compared to most other immunosuppressants when only patients with pharmaceutical immunosuppression were analyzed (Fig. S1).

Examining CAP patients only, hematologic neoplasms were present in 4% of all CAPs. In fact, they accounted for > 50% of immunocompromising factors in CAP patients followed by chronic steroid use with PDDE ≥ 10 mg (17.6%). In contrast, other authors evaluating hospitalized patients’ data only identified systemic steroids to have the highest share on immunosuppression (45%) whereas hematologic neoplasms were only found in 25% of immunocompromised CAP patients and in 2.8% of all CAPs, respectively [12, 13]. In accordance with our data this points out the notably higher and dose-dependent risk of steroid use for CAP-related complications which required hospitalization that we weighed at HR 2.7 after applying > 20 mg of PDDE.

CAP is caused by a variety of common pathogens (Woodhead et al., 1987; Almirall et al., 2000; Johansson et al., 2010; Jain et al., 2015), which are covered by empirical antibiotic treatment and were referred to as the ‘core respiratory pathogens’ in a recent widely accepted consensus statement [3]. However, previous studies suggested that immunosuppression alters the spectrum of pathogens responsible for CAP. Fungi, mycobacteria, *Pneumocystis jirovecii, Nocardia spp*. and other species are more frequently identified in immunosuppressed patients [12, 22–24]. Our recent study at a German university hospital found that > 50% of immunocmpromised patients presenting with CAP would not be adequately treated by the standard empiric therapy due to their atypical pathogen spectrum [25]. In our current investigation where we relied on ICD coding rather than validating microbiological data, CAP associated with *Aspergillus spp*., NTM and *Pneumocystis jirovecii* were coded as most common rare pathogens (Table 2). Displaying a share of 36% of 630 CAPs asociated with rare pathogens, immunosuppression was exceedingly overrepresented when compared to 7.7% in typical CAP.

Classical risk factors for typical CAP such as age and level of long-term care seem to play a minor role only when dealing with CAP associated with rare pathogens (Fig. 5). In the past there was rising concern that underlying pulmonary diseases and especially COPD put patients at an elevated risk for fungal infections like aspergillosis [26]. Additionally, they are an established risk factor for NTM. This was confirmed by our data where among comorbidities chronic pulmonary diseases were the only determinant to relevantly increase the risk to contract rare pathogen-associated CAP (HR 7.2). Beyond that, immunosuppression in general elevated the risk by more than 17-fold. HIV is by far the most considerable condition at HR 34.1. However, HIV was only diagnosed in 0.9% of immunocompromised patients compared to 39.8% exposed to relevant dosages of systemic steroids. A recent prospective study of the CAPNETZ investigator group found a relatively equal distribution of pathogens between HIV patients and controls. Typical HIV-associated species like *Pneumocystis jiroveccii* or *Aspergillus spp.* were rarely found whereas older studies found a significant number of Pneumocystis pneumonia (PCP) in HIV patients [27, 28]. At HR 8.2 systemic steroid therapy using PDDE > 20 mg poses a likewise alarming threat to contract CAP by rare pathogens. It entails a comparably high risk as solid organ transplantations (HR 8.5). Hematologic neoplasms, neutropenia or stem cell transplantations are other relevant however less hazardous and probably more widely recognized risk conditions. This correlates with the availability of guidelines concerning the implication of anti-infective prophylaxis for patients with these conditions [29–33]. In contrast, commonly applied guidelines on antimicrobial prophylactic measures in the wide range of diseases in which steroids and other immunosuppressants are administered are sparse. This might clarify the paradox of why hematologic and transplantation-associated conditions with often profound immunosuppression seem to be comparably or even less hazardous than steroid exposure alone.

None of the registered deaths due to rare pathogen-associated CAP was among HIV patients, which matches a recent nationwide German investigation which stated a significant decrease in PCP-related deaths in HIV patients [34]. This leaves other forms of immunosuppression including hematologic malignancy, stem cell or organ transplantation, antineoplastic drugs and high-dose systemic steroid therapy as most hazardous risk factors for a complicated course of CAP associated with rare pathogens.

### Strengths and limitations

We analyzed a health insurance database containing medical information encoded primarily using the German case rate-based DRG accounting system, along with other classification structures such as outpatient diagnoses, prescriptions and procedures performed. This gave us the capability to access a huge, standardized data base providing an enormous sample size for our study and avoiding selection or recall bias. We were therefore able to investigate details while providing the required power. Yet, a major limitation of working with coded data and our study design is the lack of validation of individual diagnoses. Actual medical reports, microbiological and other diagnostic findings were not provided, and medical histories could only be presumed. For example, average daily steroid doses were calculated based on dated prescriptions while the actual imposed regime remained hidden. Additionally, for CAP associated with rare pathogens we relied on ICD coding but not microbiological data, making the diagnoses uncertain. Therefore, these results should be interpreted with caution. Furthermore, we did not require diagnostic CAP confirmation by imaging. Thus, especially our ambulatory occurrence derived from ICD coding might possibly overestimate the real disease burden. However, the sensitivity and specificity of chest x-rays have been questioned [35] and in the outpatient setting treatment based on clinical signs and symptoms is a frequent approach [36]. Since we required antibiotic prescription for the case definition, the numbers most likely reflect routine practice of treatment for clinically presumed CAP in the outpatient setting in Germany. Finally, we have no data on actual causes of death, pneumonia severity other than hospitalization or mortality, applied treatment regimen and possible treatment restrictions in elderly and multimorbid patients, which might have influenced the outcome of our data.

## Conclusion

Our large, population-based study demonstrated that 6% of an adult German population showed episodes of immunosuppression within 4 years. Apart from age and level of long-term care, immunosuppression was the most relevant risk factor related to CAP and CAP-associated mortality. However, different immunosuppressive conditions cause different hazards for CAP with solid organ and stem cell transplantation, HIV infection, antineoplastic therapy as well as prescriptions of high-dose systemic steroids, rituximab or calcineurin inhibitors being associated with the highest risk. This is particularly relevant for CAP associated with rare pathogens in which HIV infection, solid organ transplantation and prescription of high dose systemic steroids are dominant risk factors. Taken together, our data provides implications for targeting preventive strategies like vaccination or specific pharmaceutic prophylactics for patients at risk.

## Electronic supplementary material

Below is the link to the electronic supplementary material.

Supplementary Material 1

## Data Availability

No datasets were generated or analysed during the current study.

## References

[1] Cheng GS, Crothers K, Evans SE, et al. Immunocompromised host pneumonia: definitions and diagnostic criteria: an official American thoracic Society Workshop Report. Ann Am Thorac Soc. 2023;20(3):341. 10.1513/ANNALSATS.202212-1019ST 36856712 10.1513/AnnalsATS.202212-1019ST PMC9993146

[2] Shea KM, Edelsberg J, Weycker D, Farkouh RA, Strutton DR, Pelton SI. Rates of pneumococcal disease in adults with chronic medical conditions. Open Forum Infect Dis. 2014;1(1). 10.1093/OFID/OFU024 10.1093/ofid/ofu024 PMC4324183 25734097

[3] Ramirez JA, Musher DM, Evans SE, et al. Treatment of community-acquired pneumonia in immunocompromised adults: a Consensus Statement regarding initial strategies. Chest. 2020;158(5):1896. 10.1016/J.CHEST.2020.05.598 32561442 10.1016/j.chest.2020.05.598 PMC7297164

[4] Kolditz M, Tesch F, Mocke L, Höffken G, Ewig S, Schmitt J. Burden and risk factors of ambulatory or hospitalized CAP: a population based cohort study. Respir Med. 2016;121:32–8. 10.1016/J.RMED.2016.10.015 27888989 10.1016/j.rmed.2016.10.015

[5] Kolditz M, Ewig S. Community-acquired pneumonia in immunocompromised adults: solid tumors might not be regarded as independent risk factors for opportunistic pathogens. Chest. 2020;158(6):2702–3. 10.1016/j.chest.2020.06.083 33280756 10.1016/j.chest.2020.06.083

[6] Wallace BI, Kenney B, Malani PN, Clauw DJ, Nallamothu BK, Waljee AK. Prevalence of Immunosuppressive Drug Use among commercially insured US adults, 2018–2019. JAMA Netw Open. 2021;4(5). 10.1001/jamanetworkopen.2021.4920 10.1001/jamanetworkopen.2021.4920 PMC8138687 34014329

[7] Shapiro BDS, Goren I, Mourad V, Cahan A. Vaccination Coverage among Immunocompromised patients in a Large Health Maintenance Organization: findings from a Novel Computerized Registry. Vaccines (Basel). 2022;10(10). 10.3390/vaccines10101654 10.3390/vaccines10101654 PMC9612260 36298519

[8] Harpaz R, Dahl RM, Dooling KL. Prevalence of immunosuppression among US adults, 2013. JAMA - J Am Med Association. 2016;316(23):2547–8. 10.1001/jama.2016.16477 10.1001/jama.2016.16477 27792809

[9] Ketkar A, Willey V, Pollack M, et al. Assessing the risk and costs of COVID-19 in immunocompromised populations in a large United States commercial insurance health plan: the EPOCH-US study. Curr Med Res Opin. 2023;39(8):1103–18. 10.1080/03007995.2023.2233819 37431293 10.1080/03007995.2023.2233819

[10] Evans RA, Dube S, Lu Y et al. Impact of COVID-19 on immunocompromised populations during the Omicron era: insights from the observational population-based INFORM study. *The Lancet Regional Health - Europe*. Published online 2023. 10.1016/j.lanepe.2023.100747 10.1016/j.lanepe.2023.100747 PMC10730312 38115964

[11] Martinson ML, Lapham J. Prevalence of Immunosuppression among US adults. JAMA Published Online Febr. 2024;15. 10.1001/jama.2023.28019 10.1001/jama.2023.28019 PMC10870224 38358771

[12] Di Pasquale MF, Sotgiu G, Gramegna A, et al. Prevalence and etiology of community-acquired Pneumonia in Immunocompromised patients. Clin Infect Dis. 2019;68(9):1482–93. 10.1093/CID/CIY723 31222287 10.1093/cid/ciy723 PMC6481991

[13] Vila-Corcoles A, Ochoa-Gondar O, Vila-Rovira A, et al. Incidence and risk of pneumococcal pneumonia in adults with distinct Underlying Medical conditions: a Population-based study. Lung. 2020;198(3):481–9. 10.1007/s00408-020-00349-y 32253492 10.1007/s00408-020-00349-y

[14] Andersen PK, Gill RD. Cox’s regression model for counting processes: a large sample study. Annals Stat. 1982;10(4):1100–20.

[15] Quan H, Sundararajan V, Halfon P, et al. Coding algorithms for defining comorbidities in ICD-9-CM and ICD-10 administrative data. Med Care. 2005;43(11):1130–9. 10.1097/01.MLR.0000182534.19832.83 16224307 10.1097/01.mlr.0000182534.19832.83

[16] Swart E, Gothe H, Geyer S, et al. Good Practice of Secondary Data Analysis (GPS): guidelines and recommendations. Gesundheitswesen. 2015;77(2):120–6. 10.1055/S-0034-1396815 25622207 10.1055/s-0034-1396815

[17] Trautmann F, Schuler M, Schmitt J. Burden of soft-tissue and bone sarcoma in routine care: estimation of incidence, prevalence and survival for health services research. Cancer Epidemiol. 2015;39(3):440–6. 10.1016/J.CANEP.2015.03.002 25801944 10.1016/j.canep.2015.03.002

[18] R Core Team. R: A Language and Environment for Statistical Computing. *R Foundation for Statistical Computing, Vienna, Austria https://*wwwR-project.org. Published online 2022.

[19] RStudio Team. RStudio: Integrated Development for R. *PBC, Boston, MA*. Published online 2020.

[20] Waljee AK, Rogers MAM, Lin P, et al. Short term use of oral corticosteroids and related harms among adults in the United States: population based cohort study. BMJ. 2017;357:j1415. 10.1136/BMJ.J1415 28404617 10.1136/bmj.j1415 PMC6284230

[21] Fardet L, Petersen I, Nazareth I. Common infections in patients prescribed systemic glucocorticoids in Primary Care: a Population-based Cohort Study. PLoS Med. 2016;13(5). 10.1371/JOURNAL.PMED.1002024 10.1371/journal.pmed.1002024 PMC4878789 27218256

[22] Garcia-Vidal C, Upton A, Kirby KA, Marr KA. Epidemiology of invasive mold infections in allogeneic stem cell transplant recipients: Biological Risk factors for infection according to Time after transplantation. Clin Infect Dis. 2008;47(8):1041–50. 10.1086/591969 18781877 10.1086/591969 PMC2668264

[23] Hammond SP, Marty FM, Bryar JM, DeAngelo DJ, Baden LR. Invasive fungal disease in patients treated for newly diagnosed acute leukemia. Am J Hematol. 2010;85(9):695–9. 10.1002/ajh.21776 20652970 10.1002/ajh.21776

[24] Moeser A, Lange C, von Lilienfeld-Toal M, Welte T, Pletz M. Pneumonien Bei Immunsupprimierten Patienten. Pneumologe (Berl). 2018;15(3):209. 10.1007/S10405-018-0174-X 32214959 10.1007/s10405-018-0174-x PMC7088144

[25] Frantz S, Schulte-Hubbert B, Halank M, Koschel D, Kolditz M. Limited prognostic accuracy of the CRB-65 and qSOFA in patients presenting with pneumonia and immunosuppression. Eur J Intern Med. 2020;81:71–7. 10.1016/J.EJIM.2020.08.006 32778480 10.1016/j.ejim.2020.08.006

[26] Bulpa P, Dive A, Sibille Y. Invasive pulmonary aspergillosis in patients with chronic obstructive pulmonary disease. Eur Respir J. 2007;30(4):782–800. 10.1183/09031936.00062206 17906086 10.1183/09031936.00062206

[27] Hoover DR, Saah AJ, Bacellar H et al. Clinical manifestations of AIDS in the era of Pneumocystis Prophylaxis. https://doi.org/101056/NEJM199312233292604. 1993;329(26):1922–6. 10.1056/NEJM199312233292604 10.1056/NEJM199312233292604 7902536

[28] Schleenvoigt BT, Ankert J, Barten-Neiner G, et al. Pathogen spectrum of community acquired pneumonia in people living with HIV (PLWH) in the German CAPNETZ-Cohort. Infection. 2023;1:1–9. 10.1007/S15010-023-02070-3/FIGURES/3 10.1007/s15010-023-02070-3 PMC10811115 37423969

[29] Stemler J, Mellinghoff SC, Khodamoradi Y, et al. Primary prophylaxis of invasive fungal diseases in patients with haematological malignancies: 2022 update of the recommendations of the Infectious Diseases Working Party (AGIHO) of the German Society for Haematology and Medical Oncology (DGHO). J Antimicrob Chemother. 2023;78(8):1813–26. 10.1093/JAC/DKAD143 37311136 10.1093/jac/dkad143 PMC10393896

[30] Henze L, Buhl C, Sandherr M, et al. Management of herpesvirus reactivations in patients with solid tumours and hematologic malignancies: update of the guidelines of the infectious diseases Working Party (AGIHO) of the German Society for Hematology and Medical Oncology (DGHO) on herpes simplex virus type 1, herpes simplex virus type 2, and varicella zoster virus. Ann Hematol. 2022;101(3):491. 10.1007/S00277-021-04746-Y 34994811 10.1007/s00277-021-04746-y PMC8810475

[31] Classen AY, Henze L, von Lilienfeld-Toal M, et al. Primary prophylaxis of bacterial infections and pneumocystis jirovecii pneumonia in patients with hematologic malignancies and solid tumors: 2020 updated guidelines of the Infectious Diseases Working Party of the German Society of Hematology and Medical Oncology (AGIHO/DGHO). Ann Hematol. 2021;100(6):1603. 10.1007/S00277-021-04452-9 33846857 10.1007/s00277-021-04452-9 PMC8116237

[32] Kasiske BL, Zeier MG, Chapman JR, et al. KDIGO clinical practice guideline for the care of kidney transplant recipients: a summary. Kidney Int. 2010;77(4):299–311. 10.1038/KI.2009.377 19847156 10.1038/ki.2009.377

[33] Maertens J, Cesaro S, Maschmeyer G, et al. ECIL guidelines for preventing Pneumocystis Jirovecii pneumonia in patients with haematological malignancies and stem cell transplant recipients. J Antimicrob Chemother. 2016;71(9):2397–404. 10.1093/JAC/DKW157 27550992 10.1093/jac/dkw157

[34] Kolbrink B, Scheikholeslami-Sabzewari J, Borzikowsky C, et al. Evolving epidemiology of pneumocystis pneumonia: findings from a longitudinal population-based study and a retrospective multi-center study in Germany. Lancet Reg Health - Europe. 2022;18. 10.1016/j.lanepe.2022.100400 10.1016/j.lanepe.2022.100400 PMC9257643 35814339

[35] Claessens YE, Debray MP, Tubach F, et al. Early chest computed Tomography scan to assist diagnosis and Guide Treatment decision for Suspected Community-acquired Pneumonia. Am J Respir Crit Care Med. 2015;192(8):974–82. 10.1164/RCCM.201501-0017OC 26168322 10.1164/rccm.201501-0017OC

[36] Van Vugt SF, Verheij TJM, De Jong PA, et al. Diagnosing pneumonia in patients with acute cough: clinical judgment compared to chest radiography. Eur Respir J. 2013;42(4):1076–82. 10.1183/09031936.00111012 23349450 10.1183/09031936.00111012

