# Supplementary material for: Epidemiology and risk factors of community-acquired pneumonia in patients with different causes of immunosuppression
Source: Infection. 2024 Jun 27;52(6):2475–86. doi: 10.1007/s15010-024-02314-w (PMC11621203; doi:10.1007/s15010-024-02314-w)

Supplement

# Epidemiology and Risk Factors of Community-Acquired Pneumonia in Patients with Different Causes of Immunosuppression

## Authors and Affiliations

Fabian Reichel^1,2*^, Falko Tesch^3^, Saskia Berger^3,4^, Martin Seifert^3^, Dirk Koschel^1,2,5^, Jochen Schmitt^3^, Martin Kolditz^1,2^

^1^ Medical Department I, Division of Pneumology, University Hospital Carl Gustav Carus, TU Dresden, Dresden, Germany

^2^ East German Lung Center / Ostdeutsches Lungenzentrum Dresden-Coswig, Germany

^3^ Center for Evidence-Based Healthcare, University Hospital and Faculty of Medicine Carl Gustav Carus at TU Dresden, Dresden, Germany

^4^ Hospital Pharmacy, University Hospital Carl Gustav Carus, Dresden, Germany

^5^ Department of Internal Medicine and Pneumology, Fachkrankenhaus Coswig, Germany

^*^ Corresponding author: [fabian.reichel@ukdd.de](mailto:fabian.reichel@ukdd.de)

**Table S1** ICD-10-GM codes of underlying immunosuppressive diseases.

| Immunosuppressive disease | Corresponding ICD-10-GM codes |
| --- | --- |
| Active hematologic neoplasm | C81.0, C81.1, C81.2, C81.3, C81.4, C81.7, C81.9, C82.0, C82.1, C82.2, C82.3, C82.4, C82.5, C82.6, C82.7, C82.9, C83.0, C83.1, C83.3, C83.5, C83.7, C83.8, C83.9, C84.0, C84.1, C84.4, C84.5, C84.6, C84.7, C84.8, C84.9, C85.1, C85.2, C85.7, C85.9, C86.0, C86.1, C86.2, C86.3, C86.4, C86.5, C86.6, C88.00, C88.20, C88.30, C88.40, C88.70, C88.90, C90.00, C90.10, C90.20, C90.30, C91.00, C91.10, C91.30, C91.40, , C91.50, C91.60, C91.70, C91.80, , C91.90, C92.00, C92.10, C92.20, C92.30, C92.40, C92.50, C92.60, C92.70, C92.80, C92.90, C93.00, C93.10, C93.30, C93.70, C93.90, C94.00, C94.20, C94.30, C94.40, C94.60, C94.70, C94.8, C95.00, C95.10, C95.70, C95.8, C95.90, C96.0, C96.2, C96.4, C96.5, C96.6, C96.7, C96.8, C96.9, D45, D46, D47.0, D47.1, D47.4, D47.5, D47.9 |
| Stem cell transplantation | T86.00, T86.01, T86.02, T86.05, T86.06, T86.07, T86.09, T86.10, Z94.80, Z94.81 |
| Solid organ transplantation | T86.11, T86.12, T86.19, T86.2, T86.3, T86.40, T86.41, T86.49, T86.81, T86.82, T86.88, Z94.0, Z94.1, Z94.2, Z94.3, Z94.4, Z94.88 |
| Neutropenia or neutrophil disorders | D70, D71, D72.0 |
| HIV infection | B20, B21, B22, B23.0, B23.8, B24 |
| Primary and humoral immune deficiency | D73.0, D76.1, D76.2, D76.3, D80.0, D80.1, D80.2, D80.3, D80.4, D80.5, D80.6, D80.8, D80.9, D81.0, D81.1, D81.2, D81.3, D81.4, D81.5, D81.6, D81.7, D81.8, D81.9, D82.0, D82.1, D82.2, D82.3, D82.4, D82.8, D82.9, D83.0, D83.1, D83.2, D83.8, D83.9, D84.0, D84.1, D84.8, D90, Q89.0 |

**Table S2** ATC and OPS codes of antineoplastic drugs.

| Substance | ATC | OPS |
| --- | --- | --- |
| Cyclophosphamide | L01AA01 |  |
| Chlorambucil | L01AA02 |  |
| Melphalan | L01AA03 |  |
| Chlormethine | L01AA04 |  |
| Ifosfamide | L01AA06 |  |
| Trofosfamide | L01AA07 |  |
| Bendamustine | L01AA09 |  |
| Busulfan | L01AB01 | 6-002.d |
| Treosulfan | L01AB02 |  |
| Thiotepa | L01AC01 | 6-007.n |
| Carmustine | L01AD01 | 6-003.3 |
| Lomustine | L01AD02 |  |
| Streptozocin | L01AD04 | 6-00b.h |
| Temozolomide | L01AX03 | 6-002.e, 6-005.c |
| Dacarbazine | L01AX04 |  |
| Methotrexate | L01BA01 |  |
| Pemetrexed | L01BA04 | 6-001.c |
| Mercaptopurine | L01BB02 |  |
| Tioguanine | L01BB03 |  |
| Cladribine | L01BB04 | 6-00a.4 |
| Fludarabine | L01BB05 |  |
| Clofarabine | L01BB06 | 6.003.j |
| Nelarabine | L01BB07 | 6-003.e |
| Cytarabine | L01BC01 | 6-002.a |
| Fluoruracil | L01BC02 |  |
| Gemcitabine | L01BC05 | 6-001.1 |
| Capecitabine | L01BC06 |  |
| Azacitidine | L01BC07 |  |
| Decitabine | L01BC08 | 6-004.4 |
| Trifluridine | L01BC59 | 6-009.n |
| Tegafur, Gimeracil, Oteracil | L01BC73 |  |
| Vinblastine | L01CA01 |  |
| Vincristine | L01CA02 |  |
| Vindesine | L01CA03 |  |
| Vinorelbine | L01CA04 |  |
| Vinflunine | L01CA05 |  |
| Etoposide | L01CB01 |  |
| Paclitaxel | L01CD01 | 6-001.f, 6-005.d |
| Docetaxel | L01CD02 | 6-002.h |
| Cabazitaxel | L01CD04 | 6-006.1 |
| Trabectedin | L01CX01 | 6-004.a |
| Dactinomycin | L01DA01 |  |
| Doxorubicin | L01DB01 | 6-001.b, 6-002.8 |
| Daunorubicin | L01DB02 |  |
| Epirubicin | L01DB03 |  |
| Idarubicin | L01DB06 |  |
| Mitoxantrone | L01DB07 |  |
| Pixantrone | L01DB11 | 6-006.e |
| Bleomycin | L01DC01 |  |
| Mitomycin | L01DC03 |  |
| Cisplatin | L01XA01 |  |
| Carboplatin | L01XA02 |  |
| Oxaliplatin | L01XA03 |  |
| Procarbazine | L01XB01 |  |
| Rituximab | L01XC02 | 6-001.h, 6-001.j |
| Trastuzumab | L01XC03 | 6-001.k |
| Gemtuzumab ozogamicin | L01XC05 | 6-00b.a |
| Cetuximab | L01XC06 | 6-001.a |
| Bevacizumab | L01XC07 | 6-002.9 |
| Panitumumab | L01XC08 | 6-004.7 |
| Ofatumumab | L01XC10 | 6-006.4 |
| Brentuximab vedotin | L01XC12 |  |
| Pertuzumab | L01XC13 | 6-007.9 |
| Trastuzumab emtansin | L01XC14 | 6-007.d |
| Obinutuzumab | L01XC15 | 6-007.j |
| Dinutuximab beta | L01XC16 | 6-009.b |
| Blinatumomab | L01XC19 | 6-008.7 |
| Ramucirumab | L01XC21 | 6-007.m |
| Necitumumab | L01XC22 | 6-009.g |
| Elotuzumab | L01XC23 | 6-009.d |
| Daratumumab | L01XC24 | 6-009.a |
| Mogamulizumab | L01XC25 |  |
| Durvalumab | L01XC27 | 6-00b.7 |
| Cemiplimab | L01XC33 |  |
| Imatinib | L01XE01 | 6-001.g |
| Gefitinib | L01XE02 |  |
| Erlotinib | L01XE03 |  |
| Sunitinib | L01XE04 | 6-003.a |
| Sorafenib | L01XE05 | 6-003.b |
| Dasatinib | L01XE06 | 6-004.3 |
| Lapatinib | L01XE07 |  |
| Nilotinib | L01XE08 | 6-004.6 |
| Temsirolismus | L01XE09 |  |
| Everolismus | L01XE10 | 6-005.8 |
| Pazopanib | L01XE11 | 6-005.a |
| Vandetanib | L01XE12 | 6-009.8 |
| Afatinib | L01XE13 |  |
| Bosutinib | L01XE14 | 6-007.4 |
| Vemurafenib | L01XE15 | 6-006.f |
| Crizotinib | L01XE16 | 6-006.c |
| Axitinib | L01XE17 | 6-006.g |
| Ruxolitinib | L01XE18 | 6-009.4 |
| Dabrafenib | L01XE23 | 6-007.5 |
| Ponatinib | L01XE24 | 6-007.b |
| Trametinib | L01XE25 | 6-009.7 |
| Cabozantinib | L01XE26 | 6-008.8 |
| Ibrutinib | L01XE27 | 6-007.e |
| Ceritinib | L01XE28 | 6-008.a |
| Lenvatinib | L01XE29 | 6-008.j |
| Nintedanib | L01XE33 |  |
| Palbociclib | L01XE33 | 6-009.j |
| Tivozanib | L01XE34 | 6-00a.j |
| Osimertinib | L01XE35 | 6-00b.f |
| Alectinib | L01XE36 | 6-00a.0 |
| Cobimetinib | L01XE38 | 6-008.c |
| Midostaurin | L01XE39 | 6-00a.b |
| Binimetinib | L01XE41 | 6-00b.2 |
| Ribociclib | L01XE42 | 6-00a.f |
| Brigatinib | L01XE43 | 6-00b.3 |
| Lorlatinib | L01XE44 |  |
| Neratinib | L01XE45 |  |
| Encorafenib | L01XE46 | 6-00b.9 |
| Dacomitinib | L01XE47 |  |
| Abemaciclib | L01XE50 | 6-00b.0 |
| Larotrectinib | L01XE53 |  |
| Gilteritinib | L01XE54 |  |
| Amsacrin | L01XX01 |  |
| Asparaginase | L01XX02 | 6-003.n, 6-003.p, 6-003.r |
| Hydroxycarbamide | L01XX05 |  |
| Pentostatin | L01XX08 |  |
| Miltefosine | L01XX09 |  |
| Estramustine | L01XX11 |  |
| Tretinoin | L01XX14 |  |
| Topotecan | L01XX17 | 6-002.4 |
| Irinotecan | L01XX19 | 6-001.3, 6-009.e |
| Alitretinoin | L01XX22 |  |
| Mitotane | L01XX23 |  |
| Pegasparase | L01XX24 |  |
| Bexarotene | L01XX25 |  |
| Arsenic trioxide | L01XX27 | 6-005.5 |
| Bortezomib | L01XX32 | 6-001.9 |
| Anagrelide | L01XX35 |  |
| Eribulin | L01XX41 | 6-006.5 |
| Panobinostat | L01XX42 | 6-009.2 |
| Vismodegib | L01XX43 |  |
| Aflibercept | L01XX44 | 6-007.2, 6-007.3 |
| Carfilzomib | L01XX45 | 6-008.9 |
| Olaparib | L01XX46 | 6-009.0 |
| Idelalisib | L01XX47 | 6-007.f |
| Sonidegib | L01XX48 |  |
| Ixazomib | L01XX50 | 6-00a.9 |
| Talimogen | L01XX51 | 6-00b.j |
| Venetoclax | L01XX52 | 6-00a.k |
| Niraparib | L01XX54 | 6-00a.c |
| Rucaparib | L01XX55 |  |
| Talazoparib | L01XX60 |  |
| Glasdegib | L01XX63 |  |
| Allogeneic T cells | L01XX90 |  |
| Tisagenlecleucel | L01XX91 |  |
| Cytarabine and Daunorubicin | L01XY01 | 6-00b.6 |
|  |  |  |
| General antineoplastic OPS Codes |  |  |
| Non-complex chemotherapy |  | 8-542 |
| Moderately complex chemotherapy |  | 8-543 |
| Highly complex chemotherapy |  | 8-544 |
| Hyperthermic chemotherapy |  | 8-546 |
| Immunotherapy using non-modified antibodies |  | 8-547.0 |
| Immunotherapy using modified antibodies |  | 8-547.1 |
| Other immunotherapy and immunosuppression |  | 8-547.3 |
| Percutaneous isolated chemotherapeutic organ perfusion |  | 8-549 |

**Table S3** ATC and OPS codes of immunosuppressants.

| Substance | ATC | OPS |
| --- | --- | --- |
| Anti-T Lymphocyte Globulin (horse/rabbit) | L04AA03, L03AA04 |  |
| Mycophenolic acid | L04AA06 |  |
| Sirolimus | L04AA10 |  |
| Leflunomide | L04AA13 |  |
| Everolimus | L04AA18 | 6-005.8 |
| Gusperimus | L04AA19 |  |
| Natalizumab | L04AA23 | 6-003.f |
| Abatacept | L04AA24 | 6-003.s,6-003.t |
| Eculizumab | L04AA25 | 6-003.h |
| Belimumab | L04AA26 | 6-006.6 |
| Fingolimod | L04AA27 |  |
| Belatacept | L04AA28 |  |
| Tofacitinib | L04AA29 |  |
| Teriflunomide | L04AA31 |  |
| Apremilast | L04AA32 |  |
| Vedolizumab | L04AA33 | 6-008.5 |
| Alemtuzumab | L04AA34, L01XC04 | 6-001.0 |
| Ocrelizumab | L04AA36 | 6-00a.e |
| Baricitinib | L04AA37 |  |
| Ozanimod | L04AA38 |  |
| Cladribine | L04AA40 | 6-00a.4 |
| Etanercept | L04AB01 | 6-002.b |
| Infliximab | L04AB02 | 6-001.e |
| Adalimumab | L04AB04 | 6-001.d |
| Certolizumab pegol | L04AB05 | 6-005.7 |
| Golimumab | L04AB06 | 6-005.2 |
| Daclizumab | L04AC01 | 6-009.9 |
| Basiliximab | L04AC02 |  |
| Anakinra | L04AC03 |  |
| Ustekinumab | L04AC05 | 6-005.j |
| Tocilizumab | L04AC07 | 6-005.m |
| Canakinumab | L04AC08 | 6-006.7 |
| Secukinumab | L04AC10 | 6-009.5 |
| Siltuximab | L04AC11 | 6-008.1 |
| Brodalumab | L04AC12 | 6-00a.3 |
| Ixekizumab | L04AC13 | 6-00a.a |
| Sarilumab | L04AC14 |  |
| Guselkumab | L04AC16 | 6-00a.7 |
| Tildrakizumab | L04AC17 | 6-00b.m |
| Ciclosporin | L04AD01 |  |
| Tacrolimus | L04AD02 |  |
| Voclosporin | L04AD03 |  |
| Azathioprine | L04AX01 |  |
| Thalidomide | L04AX02 |  |
| Methotrexate | L04AX03, M01CX01 |  |
| Lenalidomide | L04AX04 | 6-003.g |
| Pomalidomide | L04AX06 | 6-007.a |

**Table S4** ATC codes for steroids used for systemic therapy.

| Substance | Minimal dose (PDDE ≥10 mg) | ATC Code |
| --- | --- | --- |
| Prednisone | 10 mg/day | H02AB07 |
| Betamethasone | 1.4 mg/day | H02AB01, H02BX09 |
| Dexamethasone | 1.6 mg/day | H02AB02, H02BX02 |
| Fluocortolone | 10 mg/day | H02AB03, |
| Methylprednisolone | 8 mg/day | H02AB04, H02BX01 |
| Prednisolone | 10 mg/day | H02AB06, H02BX06 |
| Triamcinolone | 8 mg/day | H02AB08, H02BX08 |
| Hydrocortisone | 40 mg/day | H02AB09 |
| Cortisone | 50 mg/day | H02AB10 |
| Deﬂazacort | 12 mg/day | H02AB13 |
| Cloprednol | 7.5 mg/day | H02AB14 |

**Table S5** ICD-10-GM codes for comorbidities.

| Comorbidity group | Corresponding ICD-10-GM codes |
| --- | --- |
| chronic cardiovascular disease | I21, I22, I25.2, A52.0, I05, I06, I07, I08, I09.1, I09.8, I09.9, I11.0, I13.0, I13.2, I25.5, I27.0, I27.2, I34, I35, I36, I37, I38, I39, I42.0, I42.5, I42.6, I42.7, I42.8, I42.9, I50, P29.0, Q23.0, Q23.1, Q23.2, Q23.3, Z95.2, Z95.3, Z95.4, I70, I71, I73.1, I73.8, I73.9, I77.1, I79.0, I79.2, K55.1, K55.8, K55.9, Z95.8, Z95.9 |
| chronic cerebrovascular or neurologic disease | G10, G11, G12, G13, G20, G21, G22, G25.4, G25.5, G31.2, G31.8, G31.9, G32, G35, G36, G37, G40, G41, G45, G46, G93.1, G93.4, H34, I60, I61, I62, I63, I64, I65, I66, I67, I68, I69, R47.0, R56, F00, F01, F02, F03, F05.1, G30, G31.1, G40.1, G11.4, G80.1, G80.2, G81, G82, G83.0, G83.1, G83.2, G83.3, G83.4, G83.9 |
| chronic lung disease | J41, J42, J43, J44, J45, J46, J47, J60, J61, J62, J63, J64, J65, J66, J67, J68.4, J70.1, J70.3, J84, D86.0, D86.2 |
| chronic renal disease | I12.0, I13.1, N03.2, N03.3, N03.4, N03.5, N03.6, N03.7, N05.2, N05.3, N05.4, N05.5, N05.6, N05.7, N18, N19, N25.0, Z49.0, Z49.2, Z94.0, Z99.2 |
| chronic gastrointestinal or liver disease | K25, K26, K27, K28, K50, K51, B18, K70.0, K70.1, K70.2, K70.3, K70.4, K70.9, K71.1, K71.3, K71.4, K71.5, K71.7, K72.1, K72.9, K73, K74, K76.0, K76.2, K76.3, K76.4, K76.5, K76.6, K76.7, K76.8, K76.9, I85.0, I85.9, I86.4, I98.2, Z94.4 |
| diabetes mellitus | E10.0, E10.1, E10.6, E10.8, E10.9, E11.0, E11.1, E11.6, E11.8, E11.9, E12.0, E12.1, E12.6, E12.8, E12.9, E13.0, E13.1, E13.6, E13.8, E13.9, E14.0, E14.1, E14.6, E14.8, E14.9, E10.2, E10.3, E10.4, E10.5, E10.7, E11.2, E11.3, E11.4, E11.5, E11.7, E12.2, E12.3, E12.4, E12.5, E12.7, E13.2, E13.3, E13.4, E13.5, E13.7, E14.2, E14.3, E14.4, E14.5, E14.7 |
| chronic rheumatoid disease | M05, M06, M30, M31, M32, M33, M34, M35.0-6, M36.0, L94.0, L94.1, L94.3, M08, M12.0, M12.3, M45, M46.1, M46.8, M46.9 |
| Solid tumor | C00, C01, C02, C03, C04, C05, C06, C07, C08, C09, C10, C11, C12, C13, C14, C15, C16, C17, C18, C19, C20, C21, C22, C23, C24, C25, C26, C30, C31, C32, C33, C34, C37, C38, C39, C40, C41, C43, C45, C46, C47, C48, C49, C50, C51, C52, C53, C54, C55, C56, C57, C58, C60, C61, C62, C63, C64, C65, C66, C67, C68, C69, C70, C71, C72, C73, C74, C75, C76, C77, C78, C79, C80, C97 |

**Fig. S1** Risk factor evaluation for occurrence of CAP among patients with drug-induced immunosuppression in comparison to methotrexate. Risks were additionally adjusted for age, level of long-term care, vaccination status and type of community. 928 CAPs out of 82,252 recorded episodes of immunosuppressive therapy according to definitions II‑IV were analyzed. Confounding by antineoplastic treatment or underlying malignant disease was avoided by excluding patients treated according to definition I. As the anti-inflammatory indication of rituximab could not be distinguished from an antineoplastic use rituximab was not considered. Hazard ratios and corresponding 95 % confidence intervals were calculated using the Andersen-Gill model and are displayed as forest plot. PDDE – prednisone daily dose equivalent. * no death within 30 days of rare pathogen associated CAP diagnosis was documented for any of the HIV episodes.


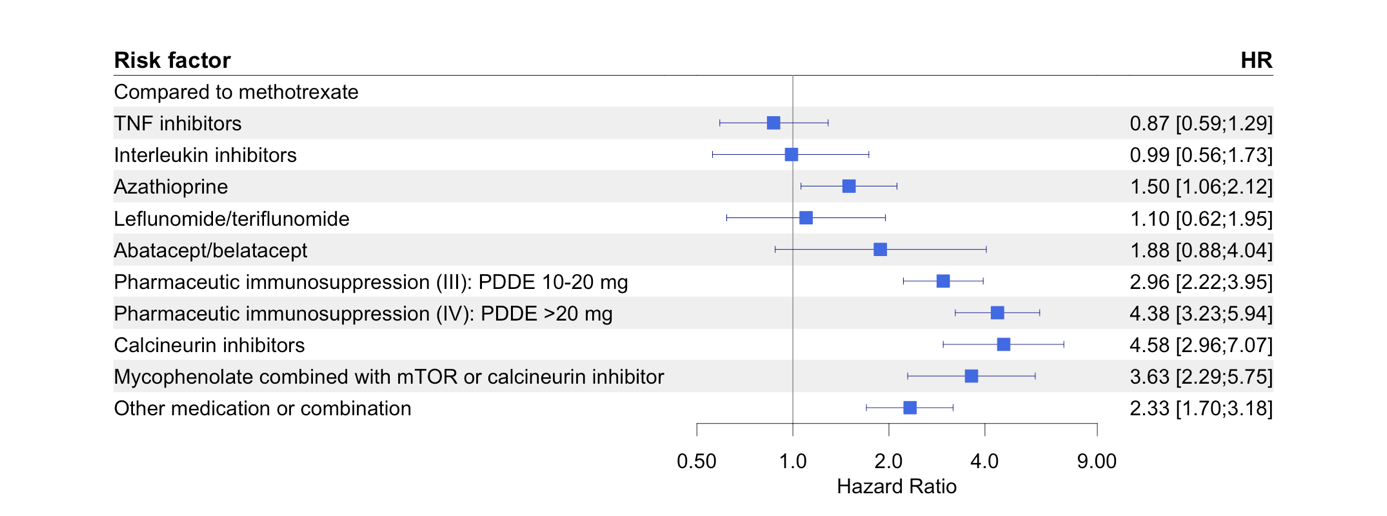


**Fig. S2** Risk factor evaluation for hospitalization of CAP. 29,916 hospitalized CAPs out of ~ 3.5 Mio. total recorded observation episodes were analyzed. Hazard ratios (HR) and corresponding 95 % confidence intervals were calculated using the Andersen-Gill model and are displayed as forest plot. PDDE – prednisone daily dose equivalent.


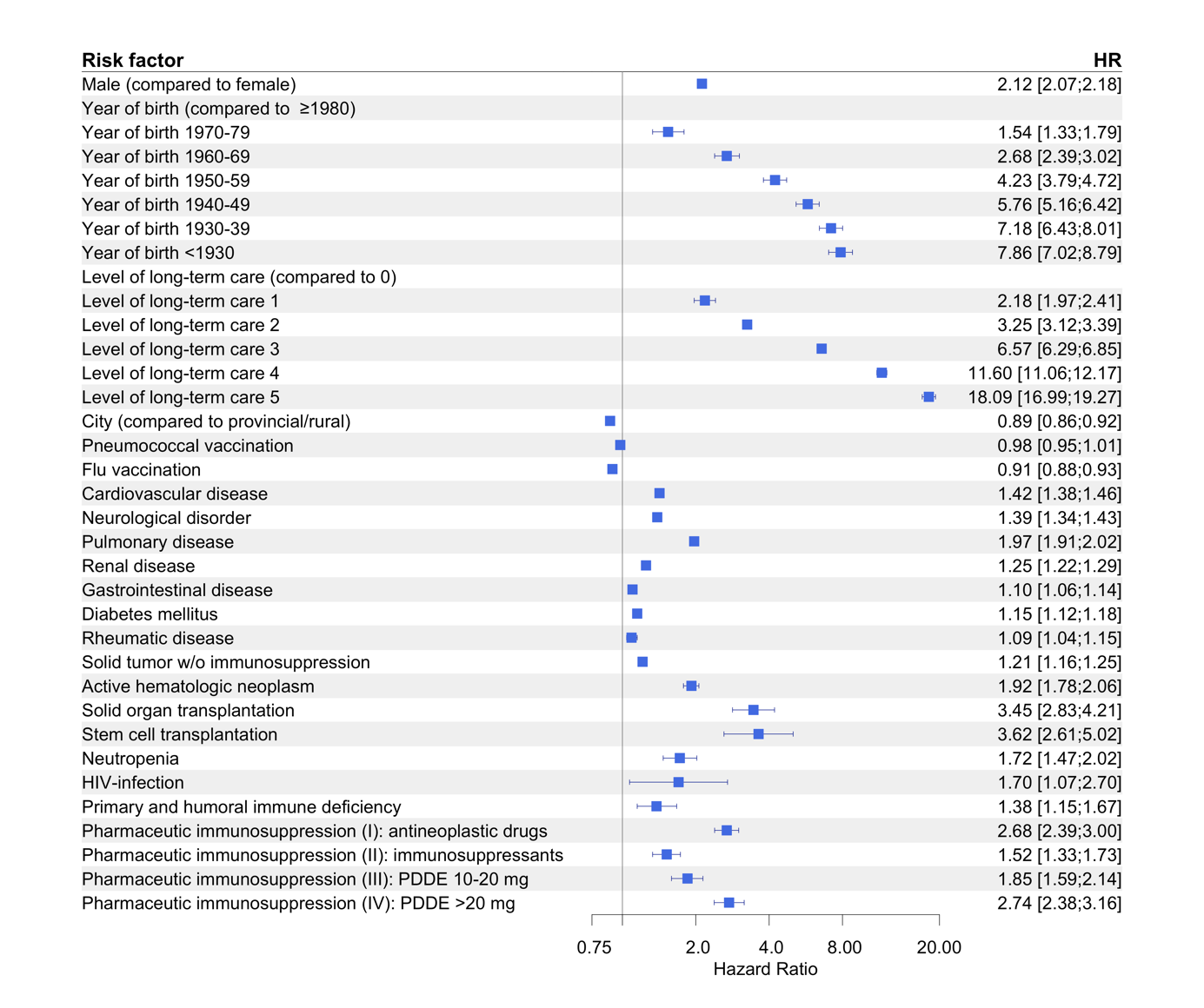


**Fig. S3** Risk factor evaluation for 30-day mortality of CAP. 7,961 CAPs that resulted in the death of the patient of any cause within 30 days after CAP diagnosis out of ~ 3.5 Mio. total recorded observation episodes were analyzed. Hazard ratios (HR) and corresponding 95 % confidence intervals were calculated using the Andersen-Gill model and are displayed as forest plot. PDDE – prednisone daily dose equivalent.


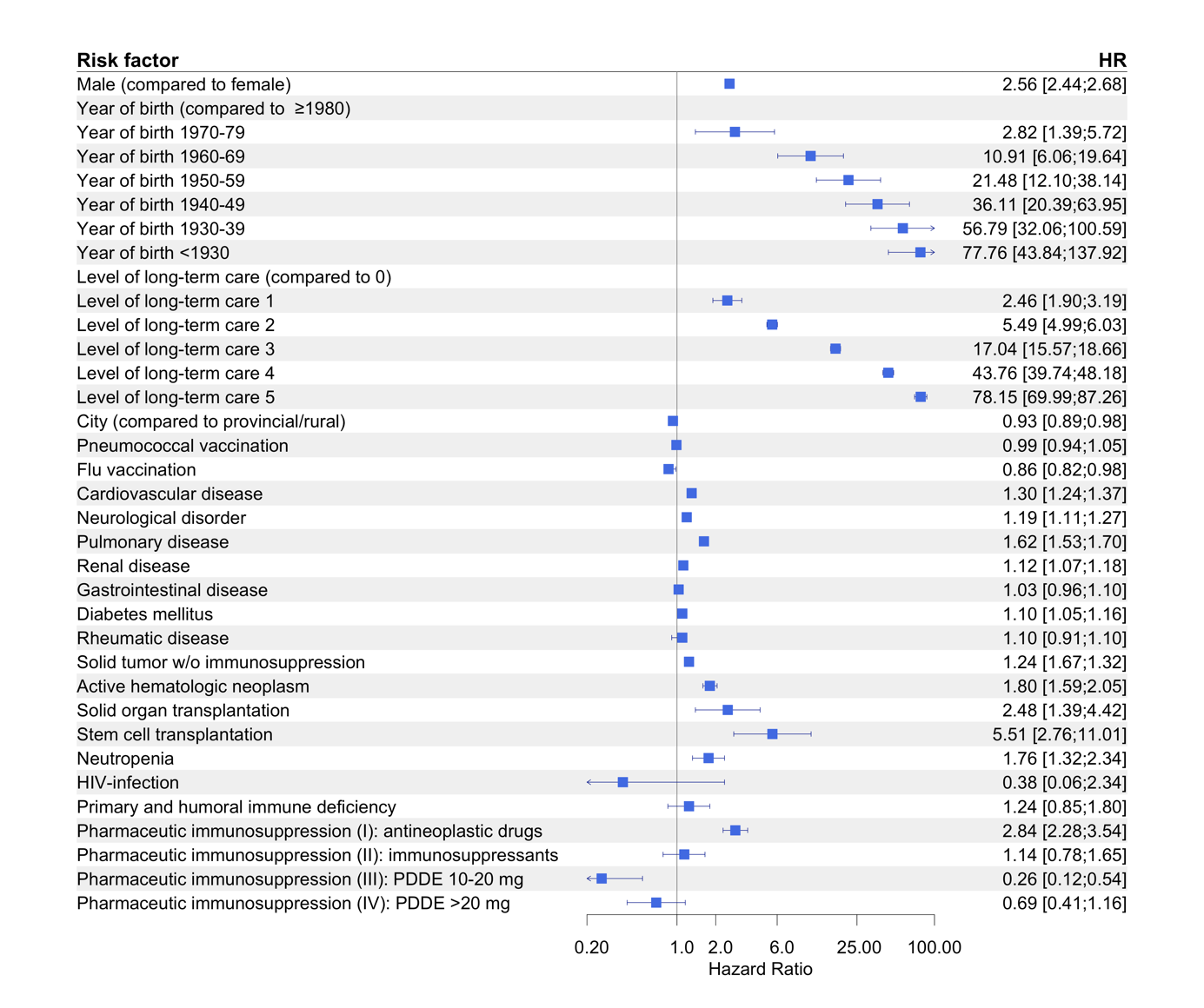


**Fig. S4** Risk factor evaluation for hospitalization of CAP associated with rare pathogens. 360 CAPs with association to rare pathogens requiring inpatient treatment out of ~ 3.5 Mio. total recorded observation episodes were analyzed. Hazard ratios (HR) and corresponding 95 % confidence intervals were calculated using the Andersen-Gill model and are displayed as forest plot. PDDE – prednisone daily dose equivalent.


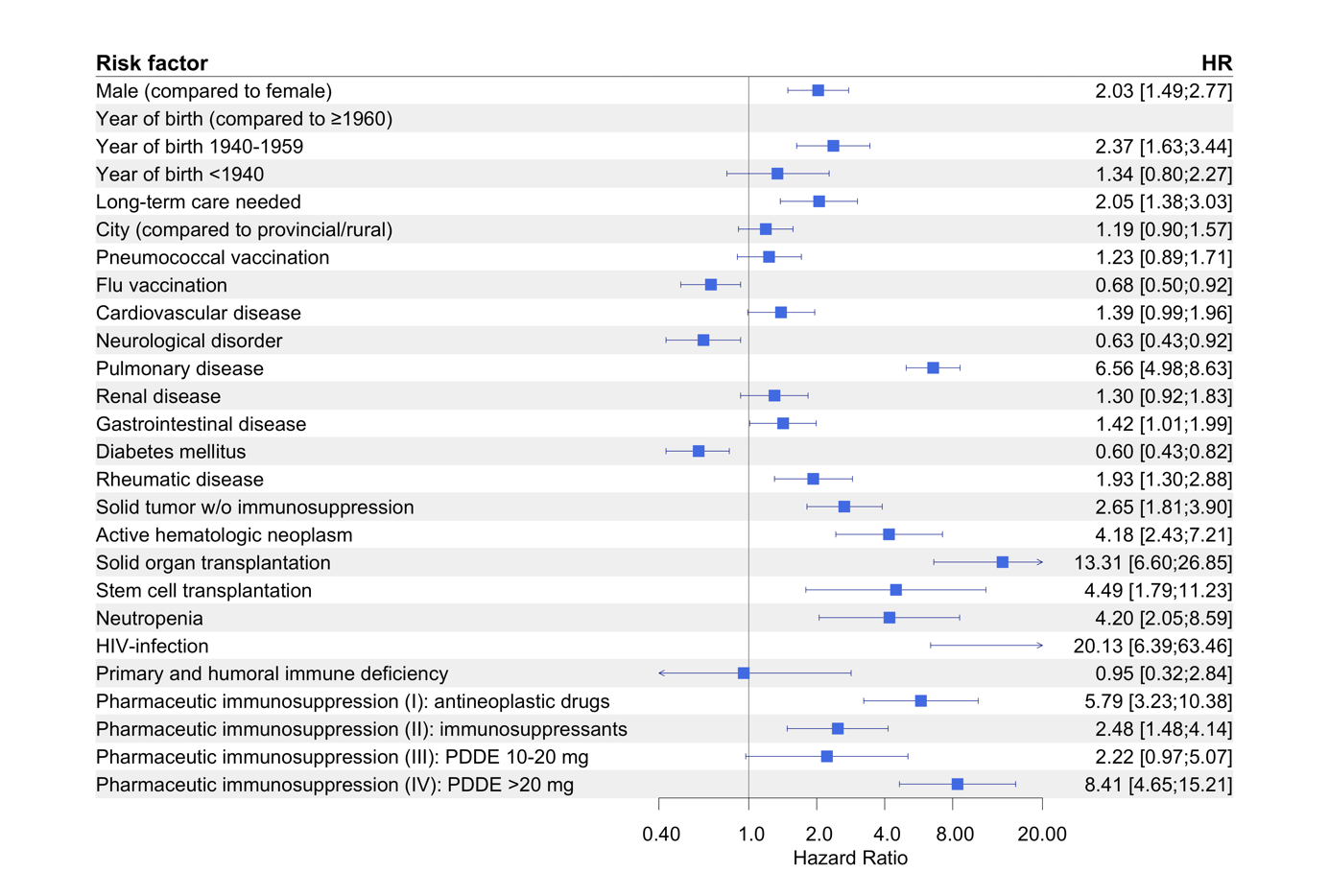


**Fig. S5** Risk factor evaluation for 30-day mortality of CAP associated with rare pathogens. 56 CAPs with association to rare pathogens with consecutively documented all-cause death within 30 days after CAP diagnosis out of ~ 3.5 Mio. total recorded observation episodes were analyzed. Hazard ratios (HR) and corresponding 95 % confidence intervals were calculated using the Andersen-Gill model and are displayed as forest plot. PDDE – prednisone daily dose equivalent.


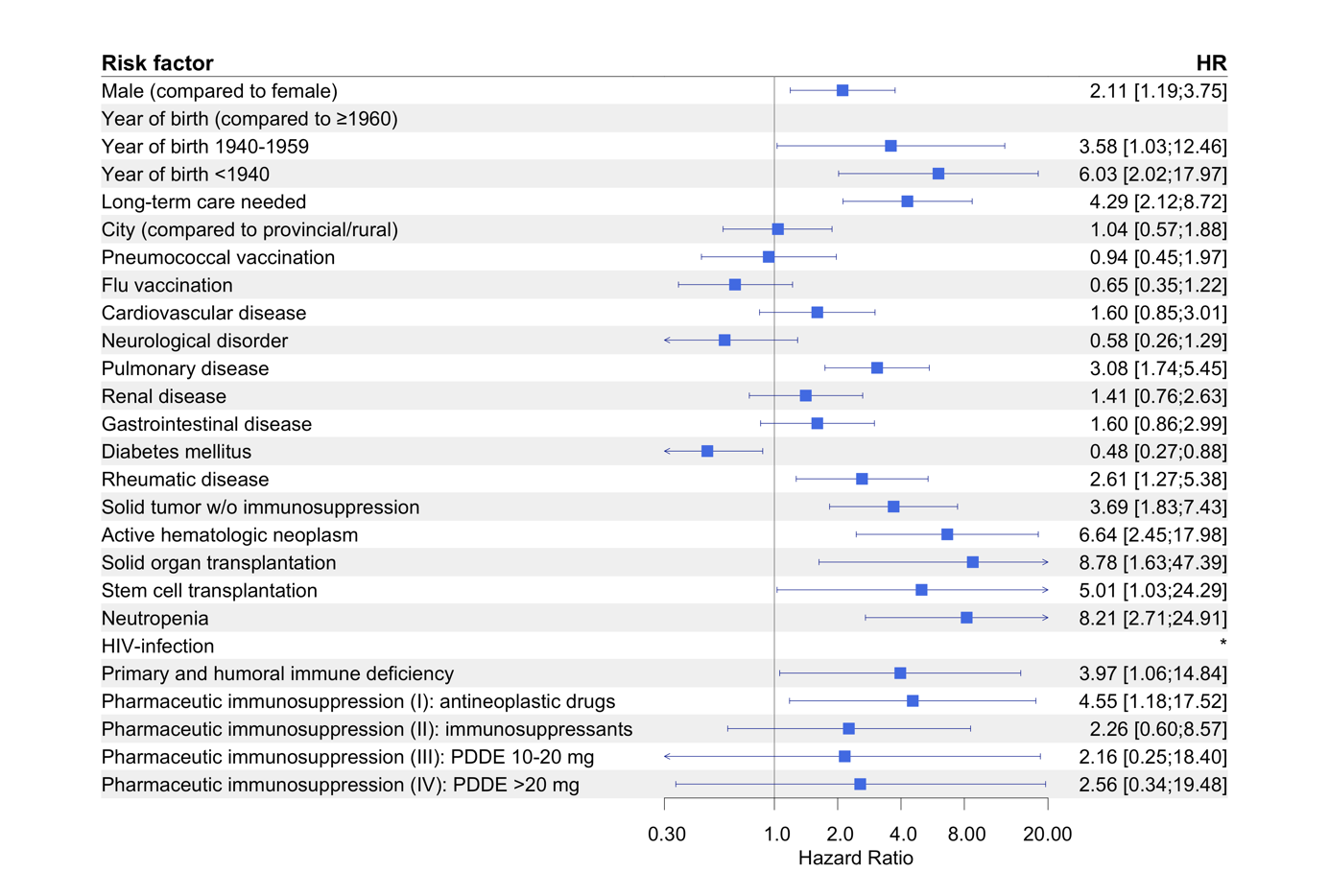

Supplement: Supplementary file 1 — Supplementary Material 1 [file 15010_2024_2314_MOESM1_ESM.docx]
